# Association between the dietary inflammatory index and disability in Japanese older people

**DOI:** 10.1017/S1368980022001604

**Published:** 2022-11

**Authors:** Momoka Masuda, Kazumi Natsuhara, Shuji Sueyoshi, Shingo Odani, Fumihiro Yagyu, Kiyoshi Tadokoro, Mayumi Ohnishi, Rieko Nakao, Chiho Goto, Masahiro Umezaki

**Affiliations:** 1 Department of Human Ecology, Graduate School of Medicine, The University of Tokyo, Tokyo 113-0033, Japan; 2 Toho University, Tokyo, Japan; 3 Kibi International University, Takahashi, Japan; 4 Chiba University, Chiba, Japan; 5 Toyo University, Tokyo, Japan; 6 Nagasaki University, Nagasaki, Japan; 7 Nagoya Bunri University, Inazawa, Japan

**Keywords:** Dietary inflammatory index, Inflammation, Diability, Older people, Japan

## Abstract

**Objectives:**

To examine the demographic and lifestyle characteristics related to the dietary inflammatory index (DII™) score and to evaluate the association between DII score and disability among older people in Japan.

**Design:**

Cross-sectional design. The DII score was calculated from nutrient intake information obtained from a FFQ. Disability was assessed using the Tokyo Metropolitan Institute of Gerontology Index of Competence questionnaire. Overall disability and disability in each component of everyday competence, that is, instrumental activities of daily living (IADL), intellectual activities and social participation, were assessed. Those with a deficit in one or more activities were defined as disabled.

**Setting:**

Five non-urban areas in Japan.

**Participants:**

A total of 1642 Japanese older people aged 65 years or older.

**Results:**

Women, residents of Oga-shi, and those with a higher education and greater frequency of shopping followed a more anti-inflammatory diet, while those living alone and residents of Minamiawaji-shi had higher dietary inflammation. A pro-inflammatory diet was associated with higher odds of overall disability and disability in each component of competence: overall disability, OR (95 % CI) = 1·26 (1·16, 1·36); IADL disability, OR (95 % CI) = 1·16 (1·07, 1·26); disability in intellectual activities, OR (95 % CI): 1·30 (1·20, 1·40); and disability in social participation, OR (95 % CI) = 1·20 (1·11, 1·29).

**Conclusions:**

Sex, living alone, education, frequency of shopping and area of residence were shown to be determinants of DII score in Japanese older people. DII score was positively associated with disability.

Japan, known for its longevity, has the largest proportion of older people in the world, with 28 % of the population aged 65 years or older in 2019^(1)^. In ageing populations, the gap between life expectancy and healthy life expectancy is a major problem. In Japan, this gap was reported to be as large as 8·84 years in men and 12·35 years in women in 2016^(2)^. These data indicate that Japanese older people may spend approximately 10 years in an unhealthy state, possibly deprived of independence in daily activities, which could be detrimental to their well-being. Therefore, it is crucial to reduce this gap and prolong the period in which older people can live without external care.

Everyday competence is a concept that describes an individual’s ability to live independently. Lawton introduced seven sublevels of competence^(3)^: life maintenance, functional health, perception and cognition, physical self-maintenance, instrumental self-maintenance, effectance, and social role (online Supplementary Table 1). These sublevels are continuous in the order of complexity. The first three levels, that is, life maintenance, functional health, and perception and cognition, refer to the most basic physical and cognitive functions indispensable for living. Physical self-maintenance corresponds to the well-known basic activities of daily living (ADL), that is, the tasks essential for living that healthy individuals can carry out on their own. Basic ADL consists of the following six categories: ambulating, feeding, personal hygiene, dressing, continence, and toileting and can be measured by the Katz Index^(4)^. The subsequent sublevel of competence, instrumental self-maintenance, represents instrumental activities of daily living (IADL). IADL are essential activities that require more complex physical or cognitive abilities, such as shopping, taking medications, and using the telephone and can be measured by the Lawton IADL scale^(5)^. Basic ADL and IADL form the foundation of the capacity for carrying out more complex sublevels of competence, that is, effectance, defined as the capacity to perform intellectual activities, and social role, which refers to the ability to engage in social interaction.

The Tokyo Metropolitan Institute of Gerontology Index of Competence (TMIG-IC) was developed based on Lawton’s model of competence to measure the everyday competence of Japanese older people^(6)^. A higher score in TMIG-IC indicates that an individual can live without external care support, while a lower score indicates disability due to physical and cognitive decline. Being capable of these fundamental skills is crucial for the well-being of older people.

Chronic inflammation has been proposed as one of the biochemical mechanisms underlying the processes of age-related impairment of physical and cognitive function, which are the bases of everyday competence^(7,8)^. Although the whole mechanism has yet to be elucidated, it has been suggested that pro-inflammatory cytokines may decrease muscle mass by suppressing protein synthesis and promoting protein degradation, which may result in physical disabilities^(9,10)^. It is also known that some inflammatory cytokines can pass the blood–brain barrier and elevate neuroinflammation, leading to cognitive dysfunction^(11)^.

Inflammation is closely linked to dietary characteristics. Various nutrients and food items have been reported to have the potential to either elevate or suppress inflammation through diverse mechanisms. For example, carbohydrates may cause inflammation by inducing hyperglycaemia, which causes oxidative stress^(12)^. Oxidative stress and inflammation have overlapping detrimental effects, making their individual effects undistinguishable^(13)^. Riboflavin may alleviate inflammation by altering the gut microbiota; increased faecal abundance of *Faecalibacterium prausnitzii*, a bacterium that excretes anti-inflammatory metabolites, was observed after 2-week supplementation of riboflavin, although the precise mechanism is unknown^(14)^. SFA activate the toll-like receptor signalling pathways, leading to elevated inflammation^(15)^. In addition, *n*-3 fatty acids (FA) suppress inflammation by inhibiting toll-like receptor^(16)^. Nutrients with different inflammatory potentials are consumed in various combinations in the diet. As the balance of pro- and anti-inflammatory nutrients modulates the body’s inflammatory status, the dietary inflammatory index (DII^™^)^(17)^ was developed as a composite score indicating the inflammatory potential of dietary intake.

The DII is the most widely used composite score to measure dietary inflammatory potential. The DII was first developed in 2009^(18)^ and recently improved by Shivappa *et al.*^(17)^. Briefly, the DII score is calculated based on the intakes of dietary components, for example, nutrients or food items, and their inflammatory effect scores. The inflammatory effect score has been estimated for forty-five dietary components. Previous studies have demonstrated different DII scores between dietary patterns. For example, plant-based diets (vegan, vegetarian, pesco-vegetarian, semi-vegetarian or omnivorous) have lower DII scores than diets containing meat^(19)^. A study that assessed DII scores of a Mediterranean diet and a low-fat diet, both designed for chronic heart disease patients, revealed lower DII scores in the Mediterranean diet compared to the low-fat diet^(20)^.

Physical and cognitive functions are indispensable for maintaining everyday competence. Associations between DII score and the risks of physical or cognitive impairment have been reported^(21–24)^. In these studies, physical impairment was assessed by questionnaires and frailty tests^(21,22)^, and cognitive impairment was assessed by memory function tests^(23)^ or by interviews^(24)^. Despite the importance of such assessment of the relations between DII score and specific aspects of physical or cognitive functions, investigation of the impact of DII on everyday competence, the overall ability to live independently, is also needed.

Only two previous studies have examined the relationship between DII score and competence of older people^(25,26)^. Laclaustra *et al.*^(25)^ evaluated DII score and IADL disability for 1948 community-dwelling adults aged 60 years or older in Spain and reported higher odds of IADL disability in individuals with higher DII score (OR = 1·96; 95 % CI = 1·03, 3·86; *P* = 0·035). IADL disability was defined as a deficit in one or more of the activities included in the widely used Lawton and Brody scale^(5)^. In another study conducted in a cohort of healthy French adults (*n* 2796), participants in the lowest tertile of DII score at baseline (age 45–60 years) showed lower likelihood of having limitations in IADL, health-related limitations in social life, major chronic diseases, function-limiting pain and depressive symptoms after 13 years of follow-up^(26)^. These studies suggested that dietary inflammation may have a substantial influence on everyday competence of older adults.

The studies discussed above were conducted in European countries, and therefore questions remain about whether such associations between DII and everyday competence are also observed in non-European countries. Japan, with the world’s most aged population, is a country in which such investigations are needed. Only indirect evidence has been available in Japanese populations, for example, an association was found between DII and functional impairment defined by the disability certification of the long-term care insurance system^(27)^. Therefore, we conducted a study focusing on disability in the more complex behaviours required for everyday competence in Japan. In addition, the determination of demographic and lifestyle factors associated with dietary inflammation in Japanese populations will be useful for future public health interventions in Japan.

This study was performed to examine the demographic and lifestyle characteristics related to DII score and to evaluate the association between DII score and disability in behaviours required for everyday competence, measured by the TMIG-IC, in Japanese people aged 65 or older.

## Methods

### Participants

This study was conducted in five municipalities in Japan: Katsuura-shi, Chiba Prefecture; Minamiawaji-shi, Hyogo Prefecture; Nagasaki-shi, Nagasaki Prefecture; Oga-shi, Akita Prefecture; and Takahashi-shi, Okayama Prefecture. In all municipalities except Takahashi-shi, four to seven neighbourhood community associations (called *jitikai* or *kukai* in Japanese, the smallest administrative unit) were first selected after consultation with the staff of the local government. We attempted to include neighbourhood community associations with diverse characteristics. Potential participants of the present study were all people in the target neighbourhood community associations aged 40 years or older at the time of the survey. The lists of potential participants were provided by neighbourhood community associations or by municipality governments. A questionnaire was delivered to and collected from the potential participants by mail (Oga-shi, Nagasaki-shi) or by research assistants (Chiba-shi, Minamiawaji-shi). The questionnaire asked about dietary intake (FFQ), everyday competence (TMIG-IC) and individual characteristics, that is, sex, age, weight, height, area of residence (Katsuura-shi, Minamiawaji-shi, Nagasaki-shi, Oga-shi and Takahashi-shi), living alone (yes and no), education (≤ 9 years and ≥ 10 years), economic status (constrained, normal or good) and frequency of shopping (1–2 times per week and 3–6 times per week or more).

The study in Takahashi-shi was conducted using a different design, as the municipality government wished to sample participants from all 718 administrative units (called *chonaikai*). First, of 21 367 people 40 years or older at the time of the survey, 700 were randomly sampled. Then, one participant was randomly sampled from each *chonaikai* where no one was sampled at the first stage, making a total of 1047 potential participants.

Of 6662 potential participants on the list, 3384 provided responses to the questionnaire (response rate 50·8 %). A total of 1294 participants younger than 65 years of age were excluded. Those with any missing data in the following variables were excluded from statistical analysis: sex, age, height, weight, nutrient intake, TMIG-IC score, area of residence, living alone, education, subjective economic status and frequency of shopping. Of 3384 participants, 1642 fulfilled the eligibility criteria and were included in the analysis.

### FFQ

A short FFQ developed for Japanese adults was used to evaluate the nutrient intakes of participants^(28)^. This FFQ is based on a previous questionnaire developed by Tokudome *et al.*^(29)^ Of the 102 foods/food groups included in this FFQ, forty-seven items were chosen for the short version used in the present study. The validity of the FFQ was evaluated by the de-attenuated Pearson’s correlation coefficients between the intakes of twenty-six nutrients obtained from the FFQ and a 3-d weighted diet record^(30)^. The total numbers of nutrients with Pearson’s correlation coefficient > 0·40 were 15 for men and 12 for women (de-attenuated, log-transformed and energy-adjusted). The validity of FA intake was evaluated individually in comparison with plasma concentration of FA^(31)^. Moderate validity for *n*-3 PUFA and *n*-3 highly unsaturated FA was observed. The 1-year interval reproducibility has also been assessed for energy intake and twenty-four nutrients^(32)^. The Spearman rank correlation coefficients between nutrient intake obtained from FFQ conducted at two time points ranged from 0·56 to 0·74 in men and 0·54 to 0·73 in women. We adopted this FFQ because it was specifically designed for Japanese, it is brief and easy to answer, and its validity and reproducibility are higher than other short FFQ developed for Japanese^(33)^.

### Dietary inflammatory index score

The DII, the most widely used index of dietary inflammatory potential, was used to estimate the inflammatory potential of the diet of each participant^(17)^. Inflammatory effect scores have been estimated for forty-five ‘food parameters’ including macro- and micronutrients, key food items, and other dietary components, such as flavonoids. In the present study, the DII score for individuals was calculated based on the intake of twenty food parameters: protein, total fat, carbohydrate, Fe, *β*-carotene, vitamin A, vitamin D, vitamin E, thiamine, riboflavin, folic acid, vitamin C, saturated fat, MUFA, PUFA, cholesterol, dietary fibre, *n*-3 FA, *n*-6 FA and alcohol. The Z-score of the intake of each food parameter was computed referring to a global composite database constructed based on dietary data from eleven countries^(17)^. Subsequently, the Z-score was converted to a percentile score to minimise the effect of right-skewing. The percentile score was multiplied by the inflammatory effect score of each food parameter to calculate the food parameter-specific DII score. The sum of the food parameter-specific DII scores was taken as the overall DII score. Negative values indicated an anti-inflammatory diet, while positive values indicated a pro-inflammatory diet. In this study, the DII score was adjusted for energy intake by including energy intake as a covariate in the regression models.

Three validation studies of the DII have been conducted in Japan. Suzuki *et al.*^(34)^ reported elevated risk of having high-sensitivity C-reactive protein concentration (> 1·0 mg/l) in the highest quartile of DII compared to the lowest quartile of DII (OR = 1·32, 95 % CI = 1·01, 2·52). Yang *et al.*^(35)^ reported a positive association between DII score and log-transformed concentration of high-sensitivity C-reactive protein (*β* = 0·05, *P* < 0·01). Kotemori *et al.*^(36)^ reported a positive association of DII score with IL-6 in men (*P* = 0·02 and 0·03 for trend across quartiles of IL-6 concentration). Therefore, DII should be applicable for assessing the inflammatory potential of diet in the Japanese population.

### Tokyo Metropolitan Institute of Gerontology Index of Competence

The TMIG-IC is widely used in Japan to evaluate the ability of older people to live independently without external care^(6)^. This index was designed based on the seven hierarchical sublevels of competence introduced by Lawton^(3)^. TMIG-IC included three higher level components, that is, instrumental self-maintenance, effectance and social role, which were renamed IADL, intellectual activities and social participation, respectively^(6)^. Thirteen activities essential for daily living grouped into the above three components were evaluated by the TMIG-IC questionnaire: (IADL) going out alone by bus or train, shopping for daily necessities, preparing meal for oneself, paying bills, making deposits and withdrawals by oneself from bank deposits; (intellectual activities) filling out documents for pension, reading newspapers, reading books and magazines, showing interest in news stories or TV programmes about health; (social participation) visiting friends, having discussions with family and/or friends, visiting sick people, and conversing with young people (Table 1). The TMIG-IC score is the total number of activities an individual is capable of performing. High TMIG-IC scores indicate high competence. In this study, overall disability and disability in each individual component of competence were assessed for each participant. For overall disability, participants were classified as ‘disabled’ if they had a deficit in one or more of the activities included in the questionnaire. Disability in each component of competence was assessed in the same way: participants were classified as ‘disabled’ if they had a deficit in one or more of the activities of the corresponding component.

**Table 1:** Questionnaire of the Tokyo Metropolitan Institute of Gerontology Index of Competence (TMIG-IC)^(6)^

Instrumental activities of daily living (IADL)
1. Can you use public transportation (bus or train) by yourself?
2. Are you able to shop for daily necessities?
3. Are you able to prepare meals by yourself?
4. Are you able to pay bills?
5. Can you handle your own banking?
Intellectual activities
6. Are you able to fill out forms for your pension?
7. Do you read newspapers?
8. Do you read books or magazines?
9. Are you interested in news stories or programmes dealing with health?
Social participation
10. Do you visit the homes of friends?
11. Are you sometimes called on advice?
12. Are you able to visit sick friends?
13. Do you sometimes initiate conversations with young people?

### Statistical analysis

The factors explaining individual variation in the DII score were investigated. Two regression models were constructed between the DII score and the following variables: sex (male, female), age (years), BMI (kg/m^2^), area of residence (Katsuura-shi, Minamiawaji-shi, Nagasaki-shi, Oga-shi and Takahashi-shi), living alone (yes and no), education (≤ 9 years and ≥ 10 years), economic status (constrained, normal or good) and frequency of shopping (1–2 times per week, 3–6 times per week or more). Frequency of shopping was included as it is expected to be associated with dietary pattern^(37)^. Model 1 investigated the association between each variable and the DII score individually. Model 2 included all the above variables simultaneously as explanatory variables. Both models included energy intake (kcal) as a covariate to adjust for energy intake.

Multiple logistic regression analysis was performed to examine the associations of the DII score with overall disability and disability in each component of competence (IADL, intellectual activities and social participation) assessed by TMIG-IC. Overall disability was defined as a deficit in one or more of the activities included in the TMIG-IC questionnaire. Disability in each component of competence was defined as a deficit in one or more of the activities in the TMIG-IC questionnaire that corresponded to the component of competence of interest. Participants without any deficit in the questionnaire/corresponding part of the questionnaire were categorised as ‘healthy’. In the logistic regression, the odds of being ‘disabled’ were computed with the ‘healthy’ group as the reference. The following variables were included as covariates: sex (male, female), age (years), BMI (kg/m^2^), economic status (constrained, normal or good), education (≤ 9 years and ≥ 10 years) and energy intake (kcal).

Statistical analyses were performed using R (ver. 4.0.5; R Project for Statistical Computing). In all analyses, *P* < 0·05 was taken to indicate statistical significance.

## Results

Basic participant characteristics, DII score and disability are illustrated in Table 2. The 1642 participants consisted of 712 (43 %) men and 930 (57 %) women with a mean (sd) age of 75·6 (7·4) years, mean (sd) BMI of 22·8 (3·3) kg/m^2^ and mean (sd) DII score of –0·24 (1·50). Of the participants, 63 % were classified as having overall disability, and 31, 40, and 48 % had disabilities in IADL, intellectual activities, and social participation, respectively.

**Table 2:** Sociodemographic characteristics, dietary inflammatory index (DII) score^(17)^ and overall disability/disability in each component of competence measured by the Tokyo Metropolitan Institute of Gerontology Index of Competence (TMIG-IC)^(6)^ (*n* 1642)

	Mean	sd
Age (years)	75·6	7·4
BMI (kg/m^2^)	22·8	3·3
DII score	−0·24	1·50
	*n*	%
Sex		
Male	712	43
Female	930	57
TMIG-IC score (overall)		
Healthy	604	37
Incompetent	1038	63
TMIG-IC score (IADL)		
Healthy	1135	69
Disabled	507	31
TMIG-IC score (intellectual activity)		
Healthy	992	60
Disabled	650	40
TMIG-IC score (social participation)		
Healthy	858	52
Disabled	784	48
Area of residence		
Katsuura-shi, Chiba prefecture	289	18
Minamiawaji-shi, Hyogo prefecture	287	18
Nagasaki-shi, Nagasaki prefecture	326	20
Oga-shi, Akita prefecture	350	21
Takahashi-shi, Okayama prefecture	390	24
Living alone		
No	1342	82
Yes	300	18
Education		
≤ 9 years	694	42
≥ 10 years	948	58
Subjective economic status		
Constrained	582	35
Normal or good	1060	65
Frequency of shopping		
1–2 times per week or less	986	60
3–6 times per week or more	656	40

IADL, instrumental activities of daily living.

The results of regression analyses between participant characteristics and DII score are presented in Table 3. The following variables were associated with DII score in the model that included all variables simultaneously (Model 2): women had a lower DII score than men (*β* (95 % CI) = –0·63 (–0·79, –0·48)); residents of Oga-shi had a lower DII score than those from other regions (ref = Katsuura-shi) (*β* (95 % CI) = –0·22 (–0·44, –0·00)); residents of Minamiawaji-shi had a higher DII score than those from other regions (ref = Katsuura-shi) (*β* (95 % CI) = 0·67 (0·44, 0·90)); participants living alone had a higher DII score compared to those living with others (*β* (95 % CI) = 0·26 (0·08, 0·44)); participants with higher education had a lower DII score (*β* (95 % CI) = –0·33 (–0·48, –0·18); and participants who reported higher frequency of shopping had a lower DII score (*β* (95 % CI) = –0·21 (–0·35, –0·06)). Age, BMI and economic status were not significantly associated with DII score.

**Table 3:** Associations between the participant characteristics and dietary inflammatory index (DII) score^(17)^ (*n* 1642)

Characteristic	Model 1*		Model 2†	
	*β*	95 % CI	*β*	95 % CI
Sex				
Male (ref)				
Female	−0·61	–0·76, –0·45	−0·63	–0·79, –0·48
Age (years)	0·01	–0·00, 0·01	−0·00	–0·01, 0·01
BMI (kg/m^2^)	0·01	–0·01, 0·03	0·01	–0·01, 0·03
Area of residence				
Katsuura-shi, Chiba prefecture (ref)				
Minamiawaji-shi, Hyogo prefecture	0·64	0·40, 0·87	0·67	0·44, 0·90
Nagasaki-shi, Nagasaki prefecture	0·03	–0·20, 0·26	0·10	–0·13, 0·33
Oga-shi, Akita prefecture	−0·17	–0·40, 0·05	−0·22	–0·44, –0·00
Takahashi-shi, Okayama prefecture	0·01	–0·21, 0·23	0·07	–0·15, 0·28
Living alone				
No (ref)				
Yes	0·22	0·04, 0·41	0·26	0·08, 0·44
Education				
≤ 9 years (ref)				
≥ 10 years	−0·36	–0·51, –0·22	−0·33	–0·48, –0·18
Economic status				
Constrained (ref)				
Normal or good	−0·21	–0·36, –0·06	−0·13	–0·28, 0·01
Frequency of shopping				
1–2 times per week or less (ref)				
3–6 times per week or more	−0·17	–0·31, –0·02	−0·21	–0·35, –0·06

* A regression analysis of the association between each participant characteristic (explanatory variable) and the DII score (outcome variable) was performed. Energy intake was included as a covariate for energy adjustment.

† Multiple regression analysis of the associations between all participant characteristics (explanatory variables) and the DII score (outcome variable) was performed. Energy intake was included as a covariate for energy adjustment.

Table 4 shows the results of simple and multiple logistic regression analyses between DII score and disability. Analyses were conducted individually for overall disability and disability in each component of competence. The odds of overall disability and disability in all three components of competence were positively associated with DII score: overall disability (OR (95 % CI) = 1·26 (1·16, 1·36)); IADL disability (OR (95 % CI) = 1·16 (1·07, 1·26)); disability in intellectual activities (OR (95 % CI): 1·30 (1·20, 1·40)); and disability in social participation (OR (95 % CI) = 1·20 (1·11, 1·29)).

**Table 4:** Associations of overall disability and disability in components of everyday competence, as measured by the Tokyo Metropolitan Institute of Gerontology Index of Competence (TMIG-IC)^(6)^, with the dietary inflammatory index (DII) score^(17)^ (*n* 1642)*

	Overall		IADL		Intellectual activity		Social participation	
	OR	95 % CI	OR	95 % CI	OR	95 % CI	OR	95 % CI
DII score	1·26	1·16, 1·36	1·16	1·07, 1·26	1·30	1·20, 1·40	1·20	1·11, 1·29
Sex								
Male (ref)								
Female	0·54	0·42, 0·69	0·53	0·41, 0·68	0·90	0·70, 1·15	0·59	0·46, 0·74
Age	1·08	1·06, 1·10	1·11	1·09, 1·13	1·06	1·05, 1·08	1·07	1·06, 1·09
BMI	0·97	0·94, 1·00	0·96	0·93, 1·00	0·98	0·95, 1·02	0·97	0·94, 1·00
Education								
≤ 9 years (ref)								
≥ 10 years	0·63	0·50, 0·79	0·69	0·54, 0·88	0·42	0·34, 0·52	0·90	0·72, 1·12
Economic status								
Constrained (ref)								
Normal or good	0·61	0·48, 0·77	0·69	0·54, 0·88	0·63	0·50, 0·80	0·72	0·58, 0·90

IADL, instrumental activities of daily living.

* The associations of the DII score (explanatory variable) with overall disability, as measured by the Tokyo Metropolitan Institute of Gerontology Index of Competence (TMIG-IC), and disability in each component of competence (IADL, intellectual activity and social participation) (outcome variables) were examined individually by multiple logistic regression analysis. The following variables were included as covariates: sex, age, BMI, education, economic status and energy intake. Energy intake was included as a covariate for energy adjustment. Overall disability/disability in each component of competence was defined as a deficit in one or more of the activities included in the TMIG-IC questionnaire or the corresponding component. The reference group was not disabled; the OR represent the odds of being disabled.

## Discussion

### Summary of findings

This cross-sectional study was performed to examine demographic and lifestyle characteristics related to DII score and the association between DII score and disability in Japanese older people. Several factors were shown to be associated with DII score: women, residents of Oga-shi, those with higher education level and those with higher frequency of shopping followed a more anti-inflammatory diet, while those living alone and residents of Minamiawaji-shi had a higher level of dietary inflammation. DII score showed positive associations with overall disability and disability in all three components of competence, that is, IADL, intellectual activities and social participation.

### Demographic and lifestyle characteristics related to dietary inflammatory index score

In the present study, women consumed a more anti-inflammatory diet, as compared to men, which was consistent with previous studies^(34,35,38)^. This may be because food preference differs between sexes; Japanese women tended to report avoiding high-fat food and choosing more fruits and foods higher in fibre, as compared to men^(39)^, which may lead to differences in dietary inflammation.

Older people living alone had higher DII scores than those living with others. A systematic review reported that living alone was related to lower dietary diversity, lower consumption of fruits, vegetables, and fish, and a higher likelihood of eating an unhealthy diet^(40)^. Hanna & Collins^(40)^ discussed several possible reasons why living alone may affect diet: living alone may reduce the motivation for cooking and eating; people living alone may lack assistance in accessing food; and living alone is costly, leading to a reduction in expenses for food. These considerations may also be applicable in Japanese older adults^(41)^.

Education and frequency of shopping were both inversely associated with DII score. Education is a well-known determinant of nutritional intake. In a study conducted in Japanese adults, participants with higher education reported eating more vegetables^(42)^. Highly educated people may have more knowledge in nutrition^(43)^, and may therefore consume more fruits and vegetables^(44)^, which often have anti-inflammatory effects. In Japan, the frequency of shopping may reflect access to perishable products, such as fish and fresh produce, which are likely to be anti-inflammatory.

Area of residence was also shown to be associated with DII score in this study. Participants from Oga-shi had lower scores, while participants from Minamiawaji-shi had higher scores compared to those from other areas. There was a difference in the degree of rurality among the study areas: Oga-shi is relatively remote and rural, whereas Minamiawaji-shi is relatively urban, located close to Metropolitan Keihan. It is possible that residents of Oga-shi have a more rural diet, while residents of Minamiawaji-shi have a more urban diet. Focusing on individual nutrients, high *β*-carotene and vitamin D intakes may have led to the lower DII score in Oga-shi (online Supplementary Table 2). In Minamiawaji-shi, low intake of dietary fibre and micronutrients, such as *β*-carotene and vitamins C, E, and D, may have led to the high DII score (online Supplementary Table 2). The potential differences in DII score among regions in Japan suggest the importance of region-specific nutritional programmes.

### Dietary inflammatory index score and disability

This study revealed a positive association between DII score and disability in behaviours required for everyday competence in Japanese older people, which was consistent with previous findings reported in European countries. Laclaustra *et al.*^(25)^ reported increased risk of overall IADL disability in adults with a higher DII score, and Assmann *et al.*^(26)^ found a greater chance of a composite outcome of having chronic diseases, IADL disability, function-limiting pain, depressive symptoms and health-related limitations in social life in individuals with a high DII score. Furthermore, Assmann *et al.*^(26)^ assessed the association between dietary inflammation and the individual components of the composite outcome and showed that the risk to cognitive ability increased with increasing dietary inflammation, while no associations were observed for IADL disability, physical disability or health-related limitations in social life^(26)^. In the present study, the DII score was related to overall disability and disability in all components of competence (IADL, intellectual activities and social participation) in older people in Japan. The association between dietary inflammation and disability of older people may not be limited to European populations but may be universal to diverse populations with different diets, including the Japanese population.

Dietary inflammation may influence disability through modulating physical and cognitive functions. DII score has been reported to show positive associations with physical and cognitive impairment^(21–24)^. A pro-inflammatory diet elevates the levels of pro-inflammatory cytokines, which can decrease muscle mass and increase neuroinflammation, leading to physical and cognitive decline^(9–11)^. Impairment of these basic functions limits an individual’s ability to perform activities that require these functions, including IADL, intellectual activities and social participation. Our results were also consistent with a previous study that revealed an association between DII score and functional disability in Japanese older people^(27)^. This study measured impairment in very basic functions, while our study measured disability in more complex behaviours required for everyday competence. As both were positively associated with DII, dietary inflammation may influence both severe and early stages of disability in Japanese older people.

Several studies have suggested beneficial health effects of the Japanese diet, related to parameters of lipid metabolism^(45–47)^. It has also been reported that the level of inflammation measured by high-sensitivity C-reactive protein is low in the Japanese population compared to Western populations^(48)^. Taken together, these observations suggest that the Japanese diet has low inflammatory potential. Studies in Japan on the impact of dietary inflammation on everyday competence among older people should add significantly to the existing knowledge from Western populations, since it may indicate the effect of dietary inflammation with lower exposure levels.

### Recommendations for public health policies

Reducing dietary inflammation may be an effective measure to maintain everyday competence in Japanese older people. The nutrient that made the greatest contribution to inflammation in this study population was dietary fibre, and the nutrients that most influenced the differences in DII score between areas were vitamins A, E and D (online Supplementary Table 2). As all of these nutrients are anti-inflammatory, encouraging consumption of foods containing higher levels of these nutrients, such as fruits and vegetables, eggs, fish, and dairy products, may be beneficial for reducing dietary inflammation in Japanese older people. In addition, sex, socio-economic status, living alone and shopping frequency were also related to DII score. Nutrition education programmes targeting men, people with low socio-economic status and people living alone as well as improving food access may be effective in decreasing dietary inflammation-related disability among older people in Japan.

### Limitations and strengths

This study had some limitations. First, this was a cross-sectional study, and we could not determine the causal relationship between dietary inflammation and disability in behaviours required for everyday competence. Longitudinal studies are required to understand more fully the influence of dietary inflammation on age-related disability. Second, the level of dietary inflammation in Japanese older adults, as compared to other populations, is unknown. This information would be useful for interpreting our results. Studies with more diverse participants, such as international studies, are necessary to assess the characteristics of dietary inflammation in different populations. Third, few food parameters were used to calculate the DII score because the short FFQ used in this study could only estimate the intakes of twenty-seven nutrients. The level of dietary inflammation can be estimated more accurately if more food parameters are available. However, since major nutrients were included, the DII calculated here should reflect the inter-individual variation in dietary inflammation.

The major strength of this study was that it was conducted in Japan. As Japan has the world’s most ageing population, maintaining the competence of older people is an urgent public health issue. Such studies in Japan should significantly add to previous knowledge from European populations, as the Japanese diet has characteristics that are distinct from European diets, which may help to generalise the link between dietary inflammation and competence. Another strength was the usage of questionnaires tailored for the Japanese population, that is, the FFQ and the TMIG-IC were both developed for the Japanese population, which may have contributed to the accuracy of the results.

## Conclusion

In conclusion, sex, living alone, educational status, frequency of shopping and area of residence were associated with DII score, and DII score was positively associated with disability in behaviours required for everyday competence among Japanese older adults. Subsequent studies are required to address the causal links between dietary inflammation and the risk of disability, and to identify differences in dietary inflammation among diverse populations.

## Supplementary material

For supplementary material accompanying this paper visit https://doi.org/10.1017/S1368980022001604. click here to view supplementary material
