# Supplementary material for: Association between the dietary inflammatory index and disability in Japanese older people
Source: Public Health Nutr. 2022 Jul 28;25(11):3137–45. doi: 10.1017/S1368980022001604 (PMC9991663; doi:10.1017/S1368980022001604)
Supplement: Supplementary file 1 [file S1368980022001604sup001.docx]

| Supplementary Table 1. Seven sublevels of competence proposed by Lawton^(3)^ and corresponding indicators of competence | |
| --- | --- |
| Sublevels of competence | Indicators |
| 1. Life maintenance |  |
| 1. Functional health | Frailty tests^(21, 22)^ |
| 1. Perception and cognition | Memory function tests^(23)^ |
| 1. Physical self-maintenance | Katz index^(4)^ |
| 1. Instrumental self-maintenance | The Lawton IADL scale^(5)^, TMIG-IC^(6)^ |
| 1. Effectance | TMIG-IC^(6)^ |
| 1. Social role | TMIG-IC^(6)^ |

ADL, Activities of daily living

IADL, Instrumental activities of daily living

TMIG-IC, Tokyo Metropolitan Institute of Gerontology Index of Competence
